# Atypical pathogens in hospitalized patients with community-acquired pneumonia: a worldwide perspective

**DOI:** 10.1186/s12879-018-3565-z

**Published:** 2018-12-18

**Authors:** Andrea Gramegna, Giovanni Sotgiu, Marta Di Pasquale, Dejan Radovanovic, Silvia Terraneo, Luis F. Reyes, Ester Vendrell, Joao Neves, Francesco Menzella, Francesco Blasi, Stefano Aliberti, Marcos I. Restrepo, on behalf of the GLIMP Study Group

**Affiliations:** 1 Department of Pathophysiology and Transplantation, University of Milan, Internal Medicine Department, Respiratory unit and Adult Cystic Fibrosis Center, Fondazione IRCCS Ca’ Granda Ospedale Maggiore Policlinico, Via Francesco Sforza 35, 20122 Milan, Italy; 2 Clinical Epidemiology and Medical Statistics Unit, Department of Clinical and Experimental Medicine, University of Sassari, Sassari, Italy; 3 Department of Biomedical and Clinical Sciences (DIBIC), University of Milan, Section of Respiratory Diseases, Ospedale L. Sacco, ASST Fatebenefratelli-Sacco, Milan, Italy; 4 Respiratory Unit, San Paolo Hospital, Department of Medical Sciences, University of Milan, Milan, Italy; 5 Division of Pulmonary Diseases and Critical Care Medicine, The University of Texas Health Science Center at San Antonio, San Antonio, TX USA; 6 Intensive Care Unit, Hospital de Matarò, Consorci Sanitari del Maresme, Carretera de Cirera s/n, 08304 Matarò, Barcelona, Spain; 7 Internal Medicine Department, Centro Hospitalar do Porto, Porto, Portugal; 8 Department of Medical Specialties, Pneumology Unit, IRCCS Arcispedale Santa Maria Nuova, Azienda USL Reggio Emilia, Italy

**Keywords:** CAP, Atypical pathogens, Epidemiology

## Abstract

**Background:**

Empirical antibiotic coverage for atypical pathogens in community-acquired pneumonia (CAP) has long been debated, mainly because of a lack of epidemiological data. We aimed to assess both testing for atypical pathogens and their prevalence in hospitalized patients with CAP worldwide, especially in relation with disease severity.

**Methods:**

A secondary analysis of the GLIMP database, an international, multicentre, point-prevalence study of adult patients admitted for CAP in 222 hospitals across 6 continents in 2015, was performed. The study evaluated frequency of testing for atypical pathogens, including *L. pneumophila*, *M. pneumoniae*, *C. pneumoniae*, and their prevalence. Risk factors for testing and prevalence for atypical pathogens were assessed through univariate analysis.

**Results:**

Among 3702 CAP patients 1250 (33.8%) underwent at least one test for atypical pathogens. Testing varies greatly among countries and its frequency was higher in Europe than elsewhere (46.0% vs. 12.7%, respectively, *p* < 0.0001). Detection of *L. pneumophila* urinary antigen was the most common test performed worldwide (32.0%). Patients with severe CAP were less likely to be tested for both atypical pathogens considered together (30.5% vs. 35.0%, *p* = 0.009) and specifically for legionellosis (28.3% vs. 33.5%, *p* = 0.003) than the rest of the population. Similarly, *L. pneumophila* testing was lower in ICU patients. At least one atypical pathogen was isolated in 62 patients (4.7%), including *M. pneumoniae* (26/251 patients, 10.3%), *L. pneumophila* (30/1186 patients, 2.5%), and *C. pneumoniae* (8/228 patients, 3.5%). Patients with CAP due to atypical pathogens were significantly younger, showed less cardiovascular, renal, and metabolic comorbidities in comparison to adult patients hospitalized due to non-atypical pathogen CAP.

**Conclusions:**

Testing for atypical pathogens in patients admitted for CAP in poorly standardized in real life and does not mirror atypical prevalence in different settings. Further evidence on the impact of atypical pathogens, expecially in the low-income countries, is needed to guidelines implementation.

**Electronic supplementary material:**

The online version of this article (10.1186/s12879-018-3565-z) contains supplementary material, which is available to authorized users.

## Background

Community-acquired pneumonia (CAP) is a leading cause of hospitalization and death worldwide [1]. The annual estimated CAP burden in the Unites States of America (USA) accounts for more than 1.5 million adult hospitalizations and one third of hospitalized patients die within 1 year [2]. The assessment of the epidemiology of CAP-related pathogens is crucial to target appropriate empiric therapy in order to improve patients’ outcomes. The empirical coverage for atypical pathogens, including *Mycoplasma pneumoniae*, *Chlamydia pneumoniae*, and *Legionella pneumophila*, is still a matter of debate [3].

Several Authors reported on an increased trend of atypical pathogens over the last 15 years, with prevalences ranging from 6 to 40% in both Europe and USA [4]. One study performed in China showed atypical pathogens as the most frequent cause of CAP with incidence rates far exceeding *Streptococcus pneumoniae* [5]. Other studies described similar prevalences of atypical pathogens [6].

Epidemiological data are mainly based on retrospective studies or secondary analyses of local or national datasets with key design limitations, such as: 1) cultures for atypicals are rarely performed and a standardized diagnostic approach has not been adopted; 2) serology for atypical pathogens could be prescribed for epidemiological studies according to international guidelines and an all-encompassing microbiological work-up should be carried out only for hospitalized patients with severe CAP [1, 7]; 3) information on testing frequency of atypical pathogens and which population subgroups are more likely to be investigated are missing. Finally, the only published description on atypical pathogens in CAP is a secondary analysis of a retrospective database [6].

The aim of this study was to provide a real-life description of both testing frequency and prevalence of atypical pathogens in hospitalized patients with CAP worldwide, along with the evaluation of predisposing conditions for testing and risk factors for CAP caused by atypical pathogens.

## Methods

### Study design and population

The present study is based on a secondary analysis of the Global Initiative for MRSA Pneumonia (GLIMP) international database [8]. This project was not funded and relied upon voluntary site and investigator participation. The GLIMP methodology has been already published elsewhere [8]. The coordinating center (University of Texas Health at San Antonio –UT Health-, Texas, USA) received project approval by the Institutional Review Board (IRB# HSC20150184E). All participating centers followed their local law and regulations. Study participants were enrolled on a single day in the months of March, April, May, and June in 2015.

All adults (> 18 years old) hospitalized with CAP were screened for study selection. CAP was defined by the evidence of new radiological pulmonary infiltrates during the first 48 h of hospitalization and by ≥1 of the following criteria: 1) new or increased cough with/without sputum production and/or purulent respiratory secretions; 2) fever (documented rectal or oral temperature ≥ 37.8 °C) or hypothermia (documented rectal or oral temperature < 36 °C); 3) systemic inflammation (e.g., white blood cell count > 10,000/cm^3^ or < 4000/cm^3^, C-reactive protein or procalcitonin values above the local upper limit of normality). Patients hospitalized with a diagnosis of hospital-acquired and/or ventilator-associated pneumonia were excluded. Patients without any bacterial tests for atypical pathogens collected within 24 h after hospital admission were also excluded.

### Data collection and microbiology for atypical pathogens

Data were collected using REDCap™ (Research Electronic Data Capture), an electronic data capture tool hosted on the UT Health server. After study enrolment, participating centers were allowed 7 days to complete electronic data entry and confirm microbiological results.

Physicians taking care of CAP patients decided the microbiological work-up according to local standard operating procedures. Serology for atypical pathogens and urinary antigen test for *L. pneumophila* were performed by local hospital laboratories according to standard techniques. Atypical pathogens were considered: *M. pneumoniae*, *C. pneumoniae*, and *L. pneumophila*.

### Study groups

Definition of CAP caused by atypical pathogens was based on species-specific serology or urinary antigen positivity. Patients tested for atypical pathogens were defined as having at least one of the following tests: urinary antigen test for *L. pneumophila,* serology for *L. Pneumophila*, *C. pneumoniae,* and *M. pneumoniae*.

### Study definitions

CAP was deemed severe when patients were prescribed one of the following interventions: intensive care unit (ICU) admission, invasive or non-invasive mechanical ventilation, or vasopressor/inotrope administration during the first 24 h after hospital admission.

Definition of immunodepression was based on the diagnosis of ≥1of the following medical conditions in the six-month period before hospital admission: hematological malignancy, asplenia, aplastic anemia, neutropenia, long-term exposure to biological drugs or steroids or chemotherapy or immunosuppressive therapy for hematological/solid organ transplantation other than lung transplant, HIV/AIDS, and congenital or genetic immunodepression. All site investigators were provided with a protocol including the above-mentioned clinical definitions.

### Statistical analysis

Testing frequency of atypical pathogens was calculated on all CAP patients in the dataset. Prevalence of an atypical pathogen was computed based on positive results of serology and/or urinary antigen test for *L. pneumophila* performed during the first 24 h of hospital stay. Categorical variables, expressed as absolute frequencies and percentages, were compared between groups using the Chi-squared test. Regressions analyses were performed to compare prevalence and compute odds ratios (OR) with their 95% confidence interval (CI); furthermore, theywere performed to assess the relationship between atypical pathogen-related pneumonia and demographic, epidemiological, and clinical variables. Circular relation analysis using the Chi-squared test was performed to compare the prevalence between countries and continents. Statistical significance when P was < 0.05. All statistical analyses were performed with IBM SPSS, Statistics for Mac, version 22.0, and STATA 13. Prevalence maps were created using Stat Planet software.

## Results

### Testing for atypical pathogens

**Fig. 1:**
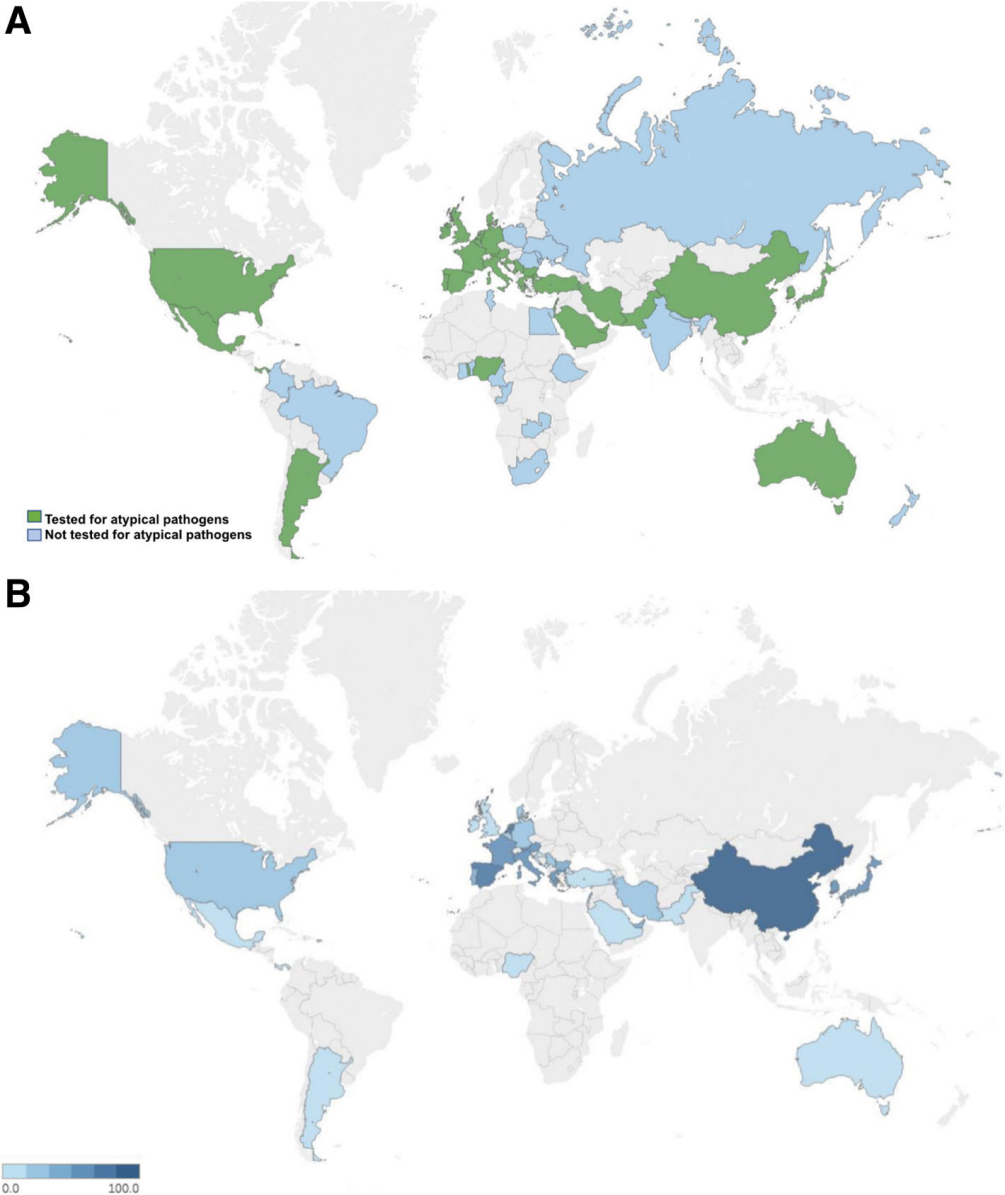
**a**) Worldwide map on testing for atypical pathogens in hospitalized patients with community-acquired pneumonia (CAP) by country. Denominator: all CAP for each country. **b**) Worldwide map on prevalence of atypical pathogens in community-acquired pneumonia (CAP) in hospitalized patients by country. Denominator: all CAP tested for atypical pathogens for each country. Figure 1 is original and it was created using Stat Planet software with the information created from the study

A total of 3702 hospitalized CAP patients were recruited in 54 countries across 6 continents. Among them, 1250 (33.8%) patients were tested for atypical pathogens: 1186 (32.0%) for *L. pneumophila* (either urinary antigen or serology), 251 (6.8%) for *M. pneumoniae* (serology), and 228 (6.1%) for *C. pneumoniae* (serology). Distribution of testing frequencies across countries is showed in Fig. 1a.

The frequency of patients tested for atypicals was significantly higher in Europe in comparison with the rest of the world (46.0% VS. 12.7%, *P* < 0.0001). The lowest testing frequency was recorded in Africa and South America (5.8 and 5.0%, respectively). The highest frequencies of patients tested for atypicals in countries enrolling > 100 CAP patients were detected in Spain (70.8%), Italy (63.8%), Portugal (43.3%), Germany (23.1%), and USA (21.4%) (Table 1). Data on testing for *L. pneumophila*, *M. pneumoniae*, and *C. pneumoniae* are reported in the additional files (Additional file 1: Table A). Detection of *L. pneumophila* urinary antigen was the most prevalent test performed worldwide (32.0%).

**Table 1:** Testing frequency for atypical pathogens (all) in hospitalized patients with community-acquired pneumonia across different continents and countries

Continent/Country	Tested/Total (%)	Rest of the world Tested/Total (%)	*P*
Europe	1078/2344 (46%)	172/1358 (12.7%)	< 0.0001
North America	105/529 (19.8%)	1145/3173 (36.1%)	< 0.0001
Asia	44/415 (10.6%)	1206/3287 (36.7%)	< 0.0001
Oceania	3/40 (7.5%)	1247/3662 (34.1%)	< 0.0001
Africa	9/156 (5.8%)	1241/3546 (35%)	< 0.0001
South America	11/218 (5%)	1239/3484 (35.6%)	< 0.0001
Spain	455/643 (70.8%)	795/3059 (26%)	< 0.0001
Italy	293/459 (63.8%)	957/3243 (29.5%)	< 0.0001
Greece	54/87 (62.1%)	1196/3615 (33.1%)	< 0.0001
France	40/66 (60.6%)	1210/3636 (33.3%)	< 0.0001
Portugal	58/134 (43.3%)	1192/3568 (33.4%)	0.018
Bulgaria	21/51 (41.2%)	1229/3651 (33.7%)	0.260
Denmark	26/89 (29.2%)	1224/3613 (33.9%)	0.358
Germany	40/173 (23.1%)	1210/3529 (34.3%)	0.002
US	102/477 (21.4%)	1148/3225 (35.6%)	< 0.0001
Serbia	10/56 (17.9%)	1240/3646 (34%)	0.011
Croatia	16/103 (15.5%)	1234/3599 (34.3%)	< 0.0001
UK	20/186 (10.8%)	1230/3516 (35%)	< 0.0001
Argentina	11/190 (5.8%)	1239/3512 (35.3%)	< 0.0001
Pakistan	6/109 (5.5%)	1244/3593 (34.6%)	< 0.0001

The frequencies of patients tested for atypical pathogens were lower among those with severe CAP in comparison with those with non-severe CAP (30.5% VS. 35.0% for atypical pathogens other than *L. pneumophila*, *P* = 0.009; 28.3% VS. 33.5% for *L. pneumophila*, *P* = 0.003). *L. pneumophila* testing was lower in ICU patients. Univariate analysis comparing characteristics of tested and non-tested patients is reported in Table 3, column A.

### Prevalence of atypical pathogens

At least one atypical pathogen was isolated in 63 (4.7%) patients out of those tested for atypicals. *L. pneumophila* was detected in 30 (2.5%), *M. pneumoniae* in 26 (10.3%), and *C. pneumoniae* in 8 (3.5%) patients. The prevalence of atypical pathogens ranged from 0.0 to 36.4% and from 0.0 to 66.7% across different continents and countries, see Fig. 1b. Italy showed the highest prevalence of atypical pathogens in comparison with the rest of the world (7.5% VS. 4.2%, *P* = 0.022), whereas Spain showed the lowest prevalence (2.2% VS. 6.5%, *P* = 0.001) (Table 2).

**Table 2:** Prevalence of atypical pathogens in hospitalized patients with community-acquired pneumonia across different continents

Continent	Tested/Total (%)	Rest of the world Tested/Total (%)	*P*
Africa	2/9 (22.2%)	60/1241 (4.8%)	0.070
Asia	2/44 (4.5%)	60/1206 (5%)	1
Europe	50/1078 (4.6%)	12/172 (7%)	0.091
North America	6/105 (5.7%)	56/1145 (4.9%)	0.922
Oceania	0/3 (0%)	62/1247 (5%)	1
South America	5/11 (45%)	57/1239 (4.6%)	0.001

Patients with CAP caused by atypical pathogens were significantly younger, showed less cardiovascular, renal, and metabolic comorbidities in comparison with patients with CAP caused by other pathogens CAP (Table 3, column B and Table 4).

**Table 3:** Clinical characteristics of tested and non-tested patients for both all atypical pathogens and L. pneumophila (column A) and of patients with community-acquired pneumonia caused and not caused by atypical pathogen (column B)

Variables	Column A			Column B		
	Tested patients (*N* = 1250)	Non-tested patients (*N* = 2452)	P	Atypical pathogen CAP (*N* = 63)	Non-atypical pathogen CAP (*N* = 1187)	*P*
Demographic characteristics						
Age, years	68 (46–75)	70 (51–81)	0.43	62 (43–72)	71 (56–81)	0.015
Male, *n* (%)	714 (57)	1459 (59)	0.83	27 (43)	687 (58)	0.027
Underweight, *n* (%)	56 (4.5)	110 (4.5)	0.36	3 (4.8)	53 (4.5)	1
Obesity, *n* (%)	208 (16.6)	369 (15)	0.21	11 (17.4)	197 (16.6)	0.811
Respiratory past medical history						
Active lung cancer, *s* (%)	27 (2.2)	82 (3.3)	0.50	0 (0)	27 (2.3)	0.64
Asthma, *n* (%)	85 (6.8)	176 (7.2)	0.73	3 (4.8)	82 (6.9)	0.79
Bronchiectasis, *n* (%)	61 (4.9)	117 (4.6)	0.87	1 (1.6)	60 (5)	0.36
Chronic aspiration, *n* (%)	71 (5.7)	186 (7.6)	0.03	0 (0)	71 (6)	0.45
COPD, *n* (%)	327 (26.2)	609 (24.8)	0.38	14 (22.2)	313 (26.3)	0.51
FEV1 ≤ 30%, *n* (%)	27 (2.2)	73 (3)	0.16	0 (0)	27 (2.2)	0.64
Current/former smoker, *n* (%)	427 (34.2)	818 (33.4)	0.63	18 (28.5)	409 (34.4)	0.382
Interstitial lung disease, *n* (%)	34 (2.7)	61 (2.5)	0.66	1 (1.6)	33 (2.8)	1
Obstructive sleep apnea, *n* (%)	51 (4.1)	79 (3.2)	0.19	0 (0)	51 (4.3)	0.17
Oxygen therapy at home, *n* (%)	83 (6.6)	141 (5.7)	0.30	4 (6.4)	79 (6.6)	1
Lung transplantation, *n* (%)	1 (0.8)	6 (0.2)	0.44	0 (0)	1 (0.8)	1
Tracheostomy, *n* (%)	15 (1)	38 (1.5)	0.45	0 (0)	15 (1.3)	1
Cardiovascular past medical history						
Arrhythmia, *n* (%)	218 (17.4)	309 (12.6)	< 0.001	11 (17.7)	207 (17.4)	0.947
Coronary artery disease, *n* (%)	178 (14.2)	345 (14.1)	0.88	3 (4.8)	175 (14.7)	0.030
Heart failure, *n* (%)	88 (7)	210 (8.2)	0.26	2 (3.2)	86 (7.2)	0.312
Hypertension, *n* (%)	183 (14.6)	323 (13)	0.14	4 (6.5)	179 (14.4)	0.790
Chronic medications						
Inhaled corticosteroids use, *n* (%)	207 (16.6)	383 (15.6)	0.47	4 (6.5)	203 (17.1)	0.028
Proton Pump Inhibitor use, *n* (%)	401 (32)	627 (25.6)	< 0.001	17 (27.4)	384 (32.3)	0.423
Statins use, *n* (%)	285 (22.8)	470 (19.2)	0.011	9 (14.5)	276 (23.2)	0.111
Steroids use, *n* (%)	86 (6.8)	208 (8.5)	0.09	4 (6.5)	82 (6.9)	1
Chronic interventions						
Enteric tube feeding, *n* (%)	11 (0.88)	41 (1.7)	0.05	0 (0)	11 (1)	1
Haemodialysis, *n* (%)	12 (1)	40 (1.6)	0.11	0 (0)	12 (1)	1
Indwelling catheter, *n* (%)	18 (1.4)	61 (2.5)	0.04	2 (3.2)	16 (1.4)	0.22
Immunosuppressive conditions						
Active solid tumour, *n* (%)	88 (7)	199 (8.1)	0.27	1 (1.6)	87 (7.3)	0.12
HIV infection, *n* (%)	28 (2.24)	95 (3.9)	0.009	2 (3.2)	26 (2.2)	0.64
AIDS, *n* (%)	15 (1.2)	50 (2)	0.08	2 (3.2)	13 (1.1)	0.16
Aplastic anaemia, *n* (%)	6 (0.3)	8 (0.3)	0.57	0 (0)	6 (0.5)	1
Asplenia, *n* (%)	6 (0.3)	6 (0.2)	0.24	0 (0)	6 (0.5)	1
Biological drug use, *n* (%)	14 (1.1)	23 (0.9)	0.60	0 (0)	14 (1.2)	1
Chemotherapy in the last 3 months, *n* (%)	48 (3.8)	97 (3.8)	0.92	1 (1.6)	47 (4)	0.51
Haematological malignancy, *n* (%)	73 (5.8)	89 (3.6)	=0.003	2 (3.2)	71 (6)	0.57
Immunocompromised patients, *n* (%)	230 (18.4)	435 (17.7)	0.62	12 (19.4)	218 (18.4)	0.84
Neutropenia, *n* (%)	13 (1.8)	35 (1.4)	0.36	0 (0)	13 (1.1)	1
Other chronic medical conditions						
Chronic renal failure, *n* (%)	144 (11.5)	256 (10.4)	0.31	2 (3.2)	142 (12)	0.036
Dementia, *n* (%)	136 (18.9)	272 (11.1)	0.87	5 (8.1)	131 (11)	0.46
Diabetes mellitus, *n* (%)	266 (21.3)	516 (21)	0.86	7 (11.3)	259 (21.8)	0.049
Liver disease, *n* (%)	59 (4.72)	81 (3.03)	0.36	4 (6.5)	55 (4.6)	0.53
Malnutrition, *n* (%)	95 (7.6)	0 (0)	0.08	4 (6.5)	91 (7.7)	1
Mental illness, *n* (%)	83 (6.6)	0 (0)	0.73	4 (6.5)	79 (6.6)	1
Prosthetic material, *n* (%)	41 (3.3)	75 (3)	0.76	1 (1.6)	40 (3.4)	0.71
Recurrent skin infections, *n* (%)	14 (1.1)	44 (1.8)	0.13	1 (1.6)	13 (1.1)	0.51
Other non-medical conditions						
Bedridden, *n* (%)	110 (8.8)	305 (12.4)	0.001	3 (4.8)	86 (7.2)	0.61
Contact sport, *n* (%)	1 (0.1)	5	0.67	0 (0)	1 (1)	1.0
Healthcare worker, *n* (%)	20 (1.6)	27 (1.1)	0.21	5 (7.9)	15 (1.3)	0.002
Homeless, *n* (%)	12 (1.8)	23 (0.9)	1.0	0 (0)	12 (1)	1
Living in crowded conditions, *n* (%)	236 (18.9)	485 (19.8)	0.54	0 (0)	9 (0.8)	1
Nursing home resident, *n* (%)	86 (6.88)	216 (8.8)	0.042	11 (17.7)	225 (18.9)	0.81
Chronic aspiration, *n* (%)	71 (5.7)	186 (7.6)	0.034	0 (0)	62 (5.3)	0.047
Previous infections/colonization						
Prior mycobacterial diseases, *n* (%)	31 (2.5)	65 (2.6)	0.82	3 (4.8)	28 (2.4)	0.19
Prior MRSA infection/colonisation, *n* (%)	30 (2.4)	56 (2.3)	0.82	0 (0)	30 (2.5)	0.39
Prior ESBL-producing bacterial infection, *n* (%)	21 (1.7)	34 (1.4)	0.48	1 (1.6)	20 (1.7)	1
Prior *Pseudomonas* spp. infection, *n* (%)	30 (2.4)	71 (2.9)	0.45	1 (1.6)	29 (2.4)	1
Current pneumonia episode						
Severe CAP, *n* (%)	314 (25.1)	716 (29.2)	0.009	21 (33)	293 (24.7)	0.103
ICU or HDU admission, *n* (%)	277 (22.2)	619 (25.2)	=0.039	18 (28)	259 (22)	0.181
Either invasive or non-invasive ventilation, *n* (%)	206 (16.5)	456 (17.9)	0.11	12 (19)	194 (16.3)	0.531
Invasive ventilation, *n* (%)	114 (9.1)	240 (9.4)	0.55	3 (4.8)	111 (9.3)	0.230
Non-invasive ventilation, *n* (%)	118 (9.4)	231 (9)	1	9 (14.3)	109 (9.1)	0.161

*CAP; Community-acquired pneumonia, MRSA; Methicillin resistant Staphylococcus aureus, COPD; Chronic obstructive pulmonary disease, FEV*_*1*_ *; Forced expiratory volume during the first second, CAD; Coronary artery disease, ESBL; extended-spectrum beta-lactamases, LRTI; lower respiratory tract infections*

**Table 4:** Protective factors for atypical pathogens in hospitalized patients with community-acquired pneumonia

	OR (95% CI)	*P*
Age	0.583 (0.350–0.973)	0.039
Cardiovascular disease	> 0.0001	< 0.0001
Diabetes mellitus	0.464 (0.207–1.043)	0.063
Chronic renal failure	0.203 (0.480–0.865)	0.031
Severe CAP	1.769 (0.516–3.073)	0.364
Mechanical ventilation	0.288 (0.810–1.031)	0.056
ICU admission	1.156 (0.311–4.294)	0.826

ICU: intensive care unit; OR: Odds ratio; CI: confidence interval

## Discussion

This secondary analysis of the GLIMP database found that only a third of patients hospitalized for CAP were tested for atypical pathogens worldwide, with a large variability among continents and countries. Patients with severe CAP were less likely to be tested for all atypical pathogens. Furthermore, *L. pneumophila* testing frequency was lower in ICU patients. Among those tested for, the prevalence of CAP caused by atypical pathogens was low. Younger age, female gender, and having a less comorbidities (cardiovascular disease, chronic renal failure) were factors associated with CAP due to atypicals.

The most frequent test for atypical pathogens performed in hospitalized patients with CAP was the *Legionella* urinary antigen (32.0%), followed by *Legionella* serology, whereas frequency of serological testing for any atypical pathogens was very low (6.8 and 6.1% for *M. pneumoniae* and *C. pneumoniae*, respectively).

However, information on molecular biology was not retrieved in the GLIMP dataset based on missing recommendations by international guidelines [1, 7]. Although molecular techniques was found helpful in the diagnosis of CAP caused by *L. pneumophila*, findings from different studies showed that single available tests were not reliable for the detection of *M. pneumoniae* and *C. pneumoniae* in CAP patients [9–11]. In addition, molecular studies carried out in large population groups found financial limitations and lack of standardization [6, 12, 13]. Finally, these results are intended to be a real-life snapshot of what it is really done in different countries worldwide; we deem that it is unrealistic a worldwide shift to PCR techniques considering that data presented here suggest that even the most common and affordable test, the urinary antigen for *Legionella*, is not routinely prescribed.

One of the major implications of a poor standardized approach for atypical pathogen testing is the wide heterogeneity across continents and countries. In Europe, almost half of the patients in the GLIMP database was investigated for atypical pathogens, thus resulting in the highest testing frequency. However, among European countries a significant variability was found. For example, the testing frequency was higher in the Mediterranean countries than in Northern Europe, ranging from 10.7% in United Kingdom to 70.8% in Spain. This significant difference may be caused by several factors, including the importance given to atypical pathogens in relation with national epidemiological reports and the lack of interest for this microbiological work-up in countries where extensive empirical therapy is routinely offered to patients. Interestingly, although large differences in frequencies of testing were found, prevalence of atypical pathogens seems to be quite similar in Europe, ranging from 1.6 to 6.5%, with the only exception of Italy and Spain.

Furthermore, our data did not suggest significant clinical differences between patients who underwent testing for atypical pathogens and those who did not. The recent guidelines for the management of CAP published by the European Respiratory Society suggest a comprehensive microbiological work-up in severe patients [1]. However, we found that severe CAP was not a relevant driver for testing. Same results were obtained for other severity indicators, such as ICU admission, invasive/non-invasive mechanical ventilation, and administration of vasopressors. The low frequency testing may be explained by the recommendation of several guidelines on broad empirical coverage in severe patients [1, 14–16]. Notably, despite cost-effectiveness and ease of use of urinary antigen test, *L. pneumophila* testing frequency was also lower in ICU patients. These data are consistent with those showed by Singanayagam who demonstrated that pneumonia severity scores, such as PSI and CURB-65, are poor predictors of microbial etiology and that atypical pathogens are more prevalent in patients with less disease severity at their presentation [17].

The present study showed that the estimated prevalence of atypical pathogens in hospitalized CAP patients is low during a non-epidemic season (i.e., from March to June). The proportional distribution was heterogeneous and the majority of the reported cases were from Europe. Inter-continent differences suggest that prevalence is lower in Africa and South America. *L. pneumophila* and *M. pneumoniae* seem to be the most frequent pathogens worldwide. The prevalence of *M. pneumoniae* is highest in South America, whereas *L. pneumophila* did show a homogeneous geographical distribution. *L. pneumophila* prevalence was similar to that recorded by Viasus (5.4% among 3934 immunocompetent hospitalized CAP patients after a 15-year study) [18]. Conversely, our data might underestimate the high incidence of legionellosis (12%) in the US population as previously reported by Vergis [19].

The CAPO database reported on a prevalence for atypical pathogens ranging from 20 to 28% across 21 countries over a five-year period (epidemic seasons included) [6]. The Authors performed a very comprehensive microbiological work-up including PCR for atypicals for the majority of CAP patients, but it is unclear the proportion of cases diagnosed by serological or molecular techniques. National and regional epidemiological reports showed a prevalence ranging from 9 to 50% [20–24]. Singanayagam and Coworkers recently published a secondary analysis of four independent prospective CAP datasets with atypical pathogens accounting for a global frequency of 14% in patients with identified microbiological positivity [17]. Interestingly, most of these studies suggested that atypical pathogens are more relevant in the outpatient population [17, 20–24].

The prevalence estimates on atypical microorganisms might be limited. Even if the combination of serology and molecular techniques was suggested to increase sensitivity, diagnostic tools only accounted on serology for atypical pathogens and urinary antigen for *Legionella* [1, 25]. Then, prevalence estimation can depend on frequency and comprehensiveness of the microbiological work-up.

Second, since patients have been enrolled on a single day in the months of March, April and May, most of data come from non-epidemic season in northern hemisphere, thus biasing a plausible estimation of atypical pathogen epidemiology.

However, the low testing frequency underscores the poor emphasis given by physicians or local health authorities to the role of atypicals. Therefore, the controversy on empiric coverage for atypical pathogens should be addressed after a more adequate description of the epidemiological burden and a sensitization of attending physicians.

Potential risk factors for atypical pathogens were also investigated. In this analysis cardiovascular disease as well as chronic renal failure act as protective factors for atypical etiology. Our understanding is that these results might be a function of age, being patients with atypical pneumonia younger than others.

## Conclusions

In conclusion, this real-life study demonstrates that testing for atypical pathogens in hospitalized patients with CAP is not routinely performed worldwide.

Testing for atypical pathogens is poorly standardized and a wide inter-country heterogeneity was found. Testing rates could not appropriately describe prevalence of atypicals in different settings. Further studies are needed to better assess the epidemiological burden and the utility of the current microbiological and clinical recommendations.

## Additional file

Additional file 1: Table A: Tables with testing frequencies for specific atypical pathogens across continents (A1: Testing frequencies for *C. pneumoniae* across continents; A2: Testing frequencies for *M. pneumoniae* across continents; A3: Testing frequencies for *L. pneumophila* across continents).- *Brief description of the data*: a table in three parts reporting data about frequency of testing for different atypical pathogens across different continents. (DOC 50 kb)

## Abbreviations

CAP: Community-acquired pneumonia
GLIMP: Global Initiative for MRSA Pneumonia
ICU: Intensive care unit
