# Correction to: Atypical pathogens in hospitalized patients with community-acquired pneumonia: a worldwide perspective

**DOI:** 10.1186/s12879-020-05472-y

**Published:** 2020-10-13

**Authors:** Andrea Gramegna, Giovanni Sotgiu, Marta Di Pasquale, Dejan Radovanovic, Silvia Terraneo, Luis F. Reyes, Ester Vendrell, Joao Neves, Francesco Menzella, Francesco Blasi, Stefano Aliberti, Marcos I. Restrepo, on behalf of the GLIMP Study Group, Patricia Karina Aruj, Silvia Attorri, Enrique Barimboim, Juan Pablo Caeiro, Victor Hugo Cambursano, Adrian Ceccato, Julio Chertcoff, Florencia Lascar, Fernando Di Tulio, Ariel Cordon Díaz, Lautaro de Vedia, Maria Cristina Ganaha, Sandra Lambert, Gustavo Lopardo, Carlos M. Luna, Alessio Gerardo Malberti, Nora Morcillo, Silvina Tartara, Claudia Pensotti, Betiana Pereyra, Pablo Gustavo Scapellato, Juan Pablo Stagnaro, Felix Lötsch, Jean Louis Vincent, Kurt Anseeuw, Camille A. Francois, Eva Van Braeckel, Marcel Zannou Djimon, Jules Bashi, Dodo Roger, Simone Aranha Nouér, Peter Chipev, Milena Encheva, Darina Miteva, Diana Petkova, Balkissou Adamou Dodo, Mbatchou Ngahane Bertrand Hugo, Ning Shen, Jin-fu Xu, Carlos Andres Bustamante Rico, Ricardo Buitrago, Fernando Jose Pereira Paternina, Kayembe Ntumba Jean-Marie, Vesna Vladic Carevic, Marko Jakopovic, Mateja Jankovic, Zinka Matkovic, Ivan Mitrecic, Marie-Laure Bouchy Jacobsson, Anette Bro Christensen, Uff e Christian HeitmannBødtger, Christian Niels Meyer, Andreas Vestergaard Jensen, Gertrud Baunbæk-knudsen, Pelle Trier Petersen, Stine Andersen, Ibrahim El-Said Abd El-Wahhab, Nesreen Elsayed Morsy, Hanaa Shafi ek, Eman Sobh, Fabrice Bertrand, Christian Brun- Buisson, Etienne de Montmollin, Muriel Fartoukh, Jonathan Messika, Pierre Tattevin, Michael Dreher, Martin Kolditz, Matthias Meisinger, Mathias W. Pletz, Stefan Hagel, Jan Rupp, Tom Schaberg, Marc Spielmanns, Beatrice Siaw-Lartey, Katerina Dimakou, Dimosthenis Papapetrou, Evdoxia Tsigou, Mohit Bhatia, Dimitrios Ampazis, Raja Dhar, George D’Souza, Rajiv Garg, Parvaiz A. Koul, P. A. Mahesh, B. S. Jayaraj, Kiran Vishnu Narayan, Hirennappa B. Udnur, Shashi Bhaskara Krishnamurthy, Keihan Golshani, Vera M. Keatings, Ignacio Martin-Loeches, Yasmin Maor, Jacob Strahilevitz, Salvatore Battaglia, Maria Carrabba, Piero Ceriana, Marco Confalonieri, Antonella d’Arminio, Bruno Del Prato, Marino De Rosa, Riccardo Fantini, Giuseppe Fiorentino, Maria Antonia Gammino, Francesco Menzella, Giuseppe Milani, Stefano Nava, Gerardo Palmiero, Roberta Petrino, Barbra Gabrielli, Paolo Rossi, Gundi Steinhilber, Alessandro Zanforlin, Kiyoyasu Kurahashi, Zeina Aoun Bacha, Daniel Barajas Ugalde, Omar Ceballos Zuñiga, José F. Villegas, Milic Medenica, E. M. W. van de Garde, Deebya Raj Mihsra, Elliott Ridgeon, Ogonna N. O. Nwankwo, Adefuye Bolanle Olufunlola, Segaolu Olumide, Kingsley N. Ukwaja, Muhammad Irfan, Lukasz Minarowski, Skoczyński Szymo, Felipe Froes, Mariana Meireles, Cláudia Ferrão, Pedro Leuschner, João Neves, Sofi a B. Ravara, Victoria Brocovschii, Chesov Ion, Doina Rusu, Daniela Chirita, Alexei Birkun, Anna Kaluzhenina, Abdullah Almotairi, Zakeya Abdulbaqi Ali Bukhary, Jameela Edathodu, Amal Fathy, Abdullah Mushira Abdulaziz Enani, Nazik Eltayeb Mohamed, Jawed Ulhadi Memon, Nada Bogdanović, Branislava Milenkovic, Luis Borderìas, Noel Manuel Bordon Garcia, Hugo Cabello Alarcón, Catia Cilloniz, Antoni Torres, Vicens Diaz-Brito, Xavier Casas, Alicia Encabo González, Maria Luisa FernándezAlmira, Miguel Gallego, Inmaculada Gaspar-GarcÍa, Juan González del Castillo, Patricia Javaloyes Victoria, Elena Laserna Martínez, Rosa Malo de Molina, Pedro J. Marcos, Rosario Menéndez, Ana Pando-Sandoval, Cristina Prat Aymerich, Alicia Lacoma del la Torre, Ignasi García-Olivé, Jordi Rello, Silvia Moyano, Francisco Sanz, Oriol Sibila, Ana Rodrigo-Troyano, Jordi Solé-Violán, Ane Uranga, Job F. M. van Boven, Ester Vendrell Torra, Jordi Almirall Pujol, Charles Feldman, Ho Kee Yum, Arnauld Attannon Fiogbe, Ferdaous Yangui, Semra Bilaceroglu, Levent Dalar, Ufuk Yilmaz, Artemii Bogomolov, Naheed Elahi, Devesh J. Dhasmana, Rhiannon Ions, Julie Skeemer, Gerrit Woltmann, Carole Hancock, Banu Rudran, Silvia Ruiz-Buitrago, Marion Campbell, Paul Whitaker, Karen S. Allen, Veronica Brito, Jessica Dietz, Claire E. Dysart, Ricardo A. Franco-Sadud, Garnet Meie Susan M. Kellie, Mina Gaga, Stephen P. Bergin, Fayez Kheir, Mark Landmeier, Manuel Lois, Girish B. Nair, Hemali Patel, Katherine Reyes, William Rodriguez-Cintron, Shigeki Saito, Nilam J. Soni, Julio Noda, Cecilia I. Hinojosa, Stephanie M. Levine, Luis F. Angel, Antonio Anzueto, K. Scott Whitlow, John Hipskind, Kunal Sukhija, Richard G. Wunderink, Ray D. Shah, Kondwelani John Mateyo

**Affiliations:** 1 Department of Pathophysiology and Transplantation, University of Milan, Internal Medicine Department, Respiratory unit and Adult Cystic Fibrosis Center, Fondazione IRCCS Ca’ Granda Ospedale Maggiore Policlinico, Via Francesco Sforza 35, 20122 Milan, Italy; 2 Department of Clinical and Experimental Medicine, University of Sassari Clinical Epidemiology and Medical Statistics Unit, Sassari, Italy; 3 Department of Biomedical and Clinical Sciences (DIBIC), University of Milan, Section of Respiratory Diseases, Ospedale L. Sacco, ASST Fatebenefratelli-Sacco, Milan, Italy; 4 Department of Medical Sciences, University of Milan, Respiratory Unit, San Paolo Hospital, Milan, Italy; 5 Division of Pulmonary Diseases and Critical Care Medicine, The University of Texas Health Science Center at San Antonio, San Antonio, TX USA; 6 Intensive Care Unit, Hospital de Matarò, Consorci Sanitari del Maresme, Carretera de Cirera s/n, 08304 Matarò, Barcelona, Spain; 7 Internal Medicine Department, Centro Hospitalar do Porto, Porto, Portugal; 8 Department of Medical Specialties, Pneumology Unit, IRCCS Arcispedale Santa Maria Nuova, Azienda USL, Reggio Emilia, Italy

**Correction to: BMC Infect Dis 18, 677 (2018)**

**https://doi.org/10.1186/s12879-018-3565-z**

Following publication of the original article [1], the authors identified that they have been incorrectly tagged under the name of their related consortium GLIMP Study Group.

Below the GLIMP Study Group authors’ names are given:

Argentina—Patricia Karina Aruj (Department of Internal Medicine, University Hospital Alfredo Lanari, Buenos Aires, Argentina); Silvia Attorri (Hospital Luis Lagomaggiore, Mendoza, Argentina); Enrique Barimboim (Hospital Central de Mendoza, Argentina); Juan Pablo Caeiro, María I Garzón (Hospital Privado Universitario, Córdoba, Argentina); Victor Hugo Cambursano (V H Dr. Cazaux A Servicio de Neumologia, Hospital Rawson, Córdoba, Argentina); Adrian Ceccato (Hospital Nacional Prof Alejandro Posadas, Argentina); Julio Chertcoff, Florencia Lascar, Fernando Di Tulio (Critical Care Unit and Respiratory Medicine, Buenos Aires British Hospital, Buenos Aires, Argentina); Ariel Cordon Díaz (Hospital General Alvear, Ciudad, Mendoza, Argentina); Lautaro de Vedia (Respiratory Intensive Care Unit, Hospital Muñiz, Buenos Aires, Argentina); Maria Cristina Ganaha (Infectious Diseases Ward, Hospital Interzonal General de Agudos, Vicente Lopez y Planes from General Rodriguez, Buenos Aires, Argentina); Sandra Lambert (Hospital El Cruce - Alta Complejidad en Red, Argentina); Gustavo Lopardo, Hospital Bernardo Houssay, Vicente López, Argentina); Carlos M Luna (Pulmonary Medicine Division, Department of Medicine, Hospital de Clínicas, Universidad de Buenos Aires, Argentina); Alessio Gerardo Malberti (Hospital Nuestra Señora del Carmen, Argentina); Nora Morcillo and Silvina Tartara (Hospital Zonal Especializado de Agudos y Crónicos Dr. Antonio A Cetrangolo, Argentina); Claudia Pensotti (Infectious Diseases and Infection Control Department, Buenos Aires, Clinica Privada Monte Grande, Argentina); Betiana Pereyra (Hospital San Roque, Córdoba, Argentina); Pablo Gustavo Scapellato (Infectious Diseases Department, Hospital D F Santojanni, Argentina); Juan Pablo Stagnaro (HZGA Mi Pueblo, Florencio Varela, Argentina). Australia—Sonali Shah (Department of General Medicine, Austin Hospital, Heidelberg, Australia). Austria–Felix Lötsch, Florian Thalhammer (Division of Infectious Diseases and Tropical Medicine, Department of Medicine I, Medical University of Vienna, Austria). Belgium—Jean Louis Vincent (Department of Intensive Care, Erasme University Hospital, Université Libre de Bruxelles, Brussels, Belgium); Kurt Anseeuw (ZNA Campus Stuivenberg, Antwerp, Belgium); Camille A Francois (Anesthesia and critical care department, Erasme university hospital, Brussels, Belgium); Eva Van Braeckel (Department of Respiratory Medicine, Ghent University Hospital, Ghent, Belgium). Benin—Marcel Zannou Djimon, Jules Bashi, Dodo Roger (Centre Hospitalier Universitaire HKM of Cotonou, Benin). Brazil—Simone Aranha Nouér (Federal University of Rio de Janeiro, Rio de Janeiro, Brazil). Bulgaria—Peter Chipev, Milena Encheva (Clinic of Pulmonary Diseases, Military Medical Academy, Sofi a, Bulgaria); Darina Miteva (UMHAT “St. Marina”, Varna, Bulgaria); Diana Petkova (University Hospital Varna, Bulgaria. Cameroon—Balkissou Adamou Dodo (Yaounde Jamot Hospital, Yaounde, Cameroon); Mbatchou Ngahane Bertrand Hugo (Douala General Hospital, Douala, Cameroon). China—Ning Shen (Respiratory Medicine, Peking University Third Hospital, Beijing, China); Jin-fu Xu, (Department of Respiratory Medicine, Shanghai Pulmonary Hospital, Tongji University, China). Colombia— Carlos Andres Bustamante Rico, Ricardo Buitrago (Clinica Shaio, Bogota, Colombia); Fernando Jose Pereira Paternina (Las Americas Clinic, Medellin, Colombia). Congo—Kayembe Ntumba Jean-Marie (Cliniques Universitaires de Kinshasa, DR Congo). Croatia—Vesna Vladic Carevic (Interne Medicine, Dubrovnik, Croatia); Marko Jakopovic (Medical School, University of Zagreb, Department for Respiratory Diseases Jordanovac, University Hospital Centre Zagreb, Zagreb, Croatia); Mateja Jankovic (University Hospital Center Zagreb, Department for Respiratory Diseases, Zagreb, Croatia); Zinka Matkovic (University Hospital Dubrava, Zagreb, Croatia); Ivan Mitrecic (Karlovac general hospital, Karlovac, Croatia). Denmark—Marie-Laure Bouchy Jacobsson (Emergency Department in North Zealand Hospital – Hillerød, Denmark); Anette Bro Christensen (Department of Anaethesiology, Viborg Region Hospital, Denmark); Uff e Christian HeitmannBødtger (Department of Pulmonology, Naestved Hospital, Denmark); Christian Niels Meyer (Department of Internal Medicine, Roskilde Hospital, Copenhagen University Hospital, Roskilde, Denmark); Andreas Vestergaard Jensen, Gertrud Baunbæk-knudsen, Pelle Trier Petersen and Stine Andersen (Department of Lung and Infectious Diseases, Nordsjællands Hospital- Hillerød, Denmark). Egypt—Ibrahim El-Said Abd El-Wahhab (Thoracic Medicine, Faculty of Medicine, Mansoura University, Egypt); Nesreen Elsayed Morsy (Pulmonary, Critical Care and Sleep Medicine, Faculty of Medicine, Mansoura University, Mansoura, Egypt); Hanaa Shafi ek (Chest diseases department, Faculty of Medicine, Alexandria University, Egypt); Eman Sobh (Chest Diseases Department, Al-Azhar University, Cairo, Egypt). France— Fabrice Bertrand (Critical care Unit, Robert Ballanger Hospital, Aulnay sous Bois, France); Christian Brun- Buisson (Univ Hospital Henri Mondor, 94000 Créteil, France); Etienne de Montmollin (Intensive care unit, Hôpital Delafontaine, Centre hospitalier de Saint-Denis, Saint-Denis, France); Muriel Fartoukh (Unité de réanimation médico-chirurgicale, Pôle Thorax Voies aériennes, Hôpital Tenon, Groupe Hospitalier Est Parisien, France); Jonathan Messika (Publique-Hôpital de Paris, Service de Réanimation Médicochirurgicale, Hôpital Louis Mourier, Colombes, France, and Université Paris Diderot, IAME, UMR 1137, Sorbonne Paris Cité, Paris, France); Pierre Tattevin (Infectious Diseases & ICU, Pontchaillou University Hospital, Rennes, France). Germany—Michael Dreher (Department of Cardiology, Pneumology, Vascular Medicine and Intensive Care Medicine, University Hospital Aachen, Aachen, Germany); Martin Kolditz (Division of Pulmonology, Medical Department I, University Hospital Carl Gustav Carus, Technische Universität Dresden, Germany); Matthias Meisinger (Klinikum Niederlausitz GmbH, Klinik für Innere Medizin und Intensivmedizin, Senftenberg, Germany); Mathias W Pletz and Stefan Hagel (Center for Infectious Diseases and Infection Control, Jena University Hospital, Germany); Jan Rupp (Department of Molecular and Infectious Diseases, University of Lübeck, Lübeck, Germany); Tom Schaberg (Zentrum für Pneumologie, Agaplesion Diakonieklinikum Rotenburg, Germany); Marc Spielmanns (Internal Medicine Department, Pulmonary rehabilitation and Department of Health, School of Medicine, University Witten- Herdecke, St Remigius-Hospital, Leverkusen, Germany). Ghana—Beatrice Siaw-Lartey (Komfo-Anokye Teaching Hospital, Kumasi, Ghana). Greece—Katerina Dimakou (5th Rerpiratory Medicine Dpt, “SOTIRIA” Chest Hospital, Athens, Greece); Dimosthenis Papapetrou (Medical Group of Athens, Paleo Faliro Clinic, Athens, Greece); Evdoxia Tsigou and Dimitrios Ampazis, Agioi Anargiroi Hospital, Kifi ssia, Athens, Greece). India—Mohit Bhatia (S S Hospital IMS BHU Varanasi, India); Raja Dhar (Fortis Hospitals, Kolkata, India); George D’Souza (Department of Pulmonary Medicine, St John’s Medical College Hospital, Bangalore, India); Rajiv Garg (Department of Respiratory Medicine, King George’s Medical University UP, Lucknow, India); Parvaiz A Koul (Department of Internal & Pulmonary Medicine, SheriKashmir Institute of Medical Sciences, Srinagar, India); P A Mahesh and B S Jayaraj (Department of Pulmonary Medicine, JSS Medical College, JSS University, Mysore, India); Kiran Vishnu Narayan (Pulmonary Medicine, Government Medical College Kozhikode, Kerala, India); Hirennappa B Udnur and Shashi Bhaskara Krishnamurthy (Columbia Asia Hospital, Hebbal, Bengaluru, Karnataka, India). Iran—Keihan Golshani (Isfahan University of Medical Sciences, Iran). Ireland—Vera M Keatings (Letterkenny General Hospital, Co. Donegal, Ireland); Ignacio Martin-Loeches (Multidisciplinary Intensive Care Research Organization (MICRO), St James’s University Hospital, Trinity Centre for Health Sciences Dublin, Ireland). Israel—Yasmin Maor (Infectious Disease Unit, Affi liated to Tel Aviv University, Wolfson Medical Center, Holon, Israel); Jacob Strahilevitz (Department of Clinical Microbiology & Infectious Diseases, Hadassah-Hebrew University, Jerusalem, Israel). Italy—Salvatore Battaglia (University of Palermo, Pneumologia DiBiMIS, Palermo, Italy); Maria Carrabba (Internal Medicine Department, Fondazione IRCCS Ca′ Granda Ospedale Maggiore Policlinico, Milano, Italy); Piero Ceriana (Pulmonary rehabilitation, IRCCS Fondazione Maugeri, Pavia Italy); Marco Confalonieri (Department of Pulmunology, University Hospital, Trieste, Italy); Antonella d’Arminio Monforte (Department of Health Sciences, Clinic of Infectious Disease, San Paolo Hospital, University of Milan, Italy); Bruno Del Prato (Interventional Pneumology, Hospital Antonio Cardarelli, Naples, Italy); Marino De Rosa (UOC Pneumologia San Filippo Neri ASL RM E, Rome, Italy); Riccardo Fantini (Respiratory Diseases Clinic, Policlinico di Modena, Modena, Italy); Giuseppe Fiorentino (UOC Fisiopatologia e Riabilitazione Respiratoria AO Ospedali dei Colli PO, Monaldi, Italy); Maria Antonia Gammino (Pulmonary Medicine Unit, San Martino Hospital, ASL 5 Oristano, Sardegna, Italy); Francesco Menzella (Department of Cardiac- Thoracic-Vascular and Intensive Care Medicine, Pneumology Unit, IRCCSArcispedale Santa Maria Nuova, Reggio Emilia, Italy); Giuseppe Milani (Azienda Ospedaliera Sant Anna di Como, Presidio Ospedale S Anna Nuovo, Unità Operativa di Pneumologia, Como, Italy); Stefano Nava (Alma Mater University of Bologna, DIMES, Respiratory and Critical Care Unit Sant’Orsola Malpighi Hospital, Italy); Gerardo Palmiero (Respiratory Unity, Versilia Hospital, Azienda USL 12 Viareggio, Lido di Camaiore, Lucca, Italy); Roberta Petrino and Barbra Gabrielli (Emergency Medicine Unit, S. Andrea Hospital, Vercelli, Italy); Paolo Rossi (Internal Medicine Department, Azienda Ospedaliero-Universitaria S. Maria della Misericordia, Udine, Italy); Claudio Sorino (Pulmonology Unit, AO Sant’Anna di Como, Italy); Gundi Steinhilber (Spedali Civili Brescia, UO Pneumologia e Fisiopatologia Respiratoria, Brescia, Italy); Alessandro Zanforlin (ULSS 18 Rovigo, Ospedale San Luca, Trecenta, Italy). Japan—Kiyoyasu Kurahashi (Yokohama City University Medical Center, Japan). Lebanon—Zeina Aoun Bacha (Medicine school, St Joseph University, Beyrouth, Lebanon). Mexico—Daniel Barajas Ugalde (National Institute of Respiratory Diseases, Mexico); Omar Ceballos Zuñiga (Hospital General de Mexicali, Mexicali, Baja California, Mexico); José F Villegas (Hospital Universitario Monterrey, México). Montenegro—Milic Medenica, Hospital for Lung Diseases—Brezovik, Niksic, Montenegro). Netherlands—E M W van de Garde (Dept. Clinical Pharmacy, St Antonius Hospital, Utrecht/ Nieuwegein, Netherlands). Nepal—Deebya Raj Mihsra (Internal Medicine, BP Koirala Institute of Health Sciences, Nepal); Poojan Shrestha, Oxford University Clinical Research Unit, Patan Hospital, Nepal). New Zealand—Elliott Ridgeon (Medical Research Institute of New Zealand). Nigeria—Babatunde Ishola Awokola (Department of Family Medicine & Primary Care, Lily Hospitals Limited, Warri, Nigeria); Ogonna N O Nwankwo (University of Calabar Teaching Hospital, Calabar, Nigeria); Adefuye Bolanle Olufunlola (Olabisi Onabanjo University teaching hospital, Sagamu, Ogun State, Nigeria); Segaolu Olumide (Department of Medicine, Pulmonary Unit, University College Hospital, Ibadan, Nigeria); Kingsley N Ukwaja (Department of Medicine, Federal Teaching Hospital Abakaliki, Ebonyi State, Nigeria). Pakistan—Muhammad Irfan (Section of Pulmonary and Critical Care Medicine, Department of Medicine, Aga Khan University, Karachi, Pakistan). Poland—Lukasz Minarowski (Department of Lung Diseaes and Tuberculosis, Medical University of Bialystok, Poland); Skoczyński Szymon (Department of Pneumology, School of Medicine in Katowice, Medical University of Silesia, Katowice, Institute of Occupational Medicine and Environmental Health, Sosnowiec, Poland). Portugal—Felipe Froes (Hospital Pulido Valente - CHLN, Lisboa, Portugal); Pedro Leuschner (Centro Hospitalar do Porto, Porto, Portugal); Mariana Meireles, Cláudia Ferrão, Pedro Leuschner and João Neves (Serviço de Medicina, Centro Hospitalar do Porto, Largo, Abel Salazar, Porto, Portugal); Sofi a B Ravara (Faculty of Health Sciences, University of Beira Interior); Cova da Beira Hospital Center, Covilhã, Portugal). Moldova—Victoria Brocovschii (Department of Pneumology & Allergology, State University of Medicine and Pharmacy “Nicolae Testemitanu”, Moldova); Chesov Ion (Clinic of Anesthesia and Intensive Care “Valeriu Ghrerg”, Institute of Emergency Medicine, State University of Medicine and Pharmacy “Nicolae Testemitanu”, Chisinau, Moldova); Doina Rusu (SMFU “N Testemitanu”, Chisinau, Moldova); Cristina Toma (Department of Pneumology & Allergology, State University of Medicine and Pharmacy “Nicolae Testemitanu”, Chisinau, Moldova). Romania—Daniela Chirita (Hospital Sfantul Stefan, Bucharest, Romania). Russia—Alexei Birkun (Department of Anesthesiology, Critical Care and Emergency Medicine, Medical Academy named after S I Georgievsky, Russia); Anna Kaluzhenina (Volgograd State Medical University, Russia). Saudi Arabia—Abdullah Almotairi (King Fahad medical City (KFMC), Riyadh, Saudi Arabia); Zakeya Abdulbaqi Ali Bukhary (College of Medicine, Taibah University, Medina, Saudi Arabia); Jameela Edathodu (Al Faisal University, King Faisal Specialist Hospital, Riyadh, Saudi Arabia); Amal Fathy (Pulmonary and respiratory critical care Medicine, Mansoura University Egypt, Affi liate at Taibah University, Saudi Arabia); Abdullah Mushira Abdulaziz Enani and Nazik Eltayeb Mohamed (Infectious Diseases Section, Medical Specialties Department, King Fahad Medical City, Riyadh, Saudi Arabia); Jawed Ulhadi Memon (Pulmonology Division, Department of Internal Medicine, King Fahad Hospital, Hofuf, Al Ahasa, 31982, Saudi Arabia). Serbia—Nada Bogdanović (Pulmonary department of KHC Dr. Dragiša Miš ović, Belgrade, Serbia); Branislava Milenkovic (Clinic for Pulmonary Diseases, Clinical Centre of Serbia, Faculty of Medicine, University of Belgrade, Belgrade, Serbia); Dragica Pesut (University of Belgrade School of Medicine, Teaching Hospital of Pulmonology, Clinical Centre of Serbia, Belgrade, Serbia). Spain—Luis Borderìas, Respiratoy and Sleep Unit, Hospital San Jorge, Huesca, Spain); Noel Manuel Bordon Garcia (Barcelona Policlínic and Moises Broggi Hospital at sant Joan Despí, Spain); Hugo Cabello Alarcón, Sant Hospital Seu de Urgell, Catalonia, Spain); Catia Cilloniz and Antoni Torres (Department of Pneumology, Institut Clinic del Tórax, Hospital Clinic of Barcelona - Institut d’Investigacions Biomèdiques August Pi i Sunyer (IDIBAPS), University of Barcelona, Ciber de Enfermedades Respiratorias (CIBERES), Spain); Vicens Diaz-Brito and Xavier Casas (Infectious diseases Unit and Pneumology Service, Parc Sanitari Sant Joan de Deu, Sant Boi, Barcelona, Spain); Alicia Encabo González (Hospital Complex of Pontevedra, Spain); Maria Luisa FernándezAlmira (Medicina Interna, Hospital Universitario Central de Asturias, Spain); Miguel Gallego (Department of Respiratory Medicine, Hospital de Sabadell, Institut Universitari Parc Taulí-UAB, Sabadell, Spain. CIBER de Enfermedades Respiratorias, CIBERES, Bunyola, Spain); Inmaculada Gaspar-GarcÍa (Department of Respiratory Medicine, Hospital Costa del Sol, Marbella, Málaga, Spain); Juan González del Castillo (Emergency Department, Hospital Universitario Clínico San Carlos, Madrid, Spain); Patricia Javaloyes Victoria (Hospital General Universitario de Alicante, Alicante, Spain); Elena Laserna Martínez (Hospital Mollet, Barcelona, Spain); Rosa Malo de Molina (University Hospital Puerta de Hierro Majadahonda, Madrid); Pedro J Marcos (Servicio de Neumología,Complejo Hospitalario Universitario de A Coruña CHUAC, INIBIC, Sergas,Universidade de A Coruña, Spain); Rosario Menéndez (Pneumology Service, Universitary and Polytechnic Hospital La Fe, Valencia, Spain); Ana Pando-Sandoval (Hospital Universitario Central de Asturias. Area de Gestion Clinica de Pulmon. Servicio de Neumologia, Oviedo, Spain); Cristina Prat Aymerich, Alicia Lacoma del la Torre, and Ignasi García-Olivé (Microbiology Department and Pneumology Department, Hospital Universitari Germans Trias i Pujol, Institut d’Investigació Germans Trias i Pujol, Badalona, Spain; Universitat Autònoma de Barcelona; CIBER Enfermedades Respiratorias, Instituto de Salud Carlos III, Spain); Jordi Rello and Silvia Moyano (Critical Care Department, Hospital Vall d›Hebron, Barcelona, Spain); Francisco Sanz (Servicio de Neumología, Consorci Hospital General Universitari de Valencia, Valencia, Spain); Oriol Sibila and Ana Rodrigo-Troyano (Servei de Pneumologia, Hospital de la Santa Creu i Sant Pau, IIB-Sant Pau, Barcelona, Spain); Jordi Solé-Violán (Hospital Universitario de Gran Canaria Dr. Negrín, Las Palmas de Gran Canaria, Spain); Ane Uranga (Pulmology Department, Hospital of Galdakao-Usansolo, Spain); Job FM van Boven (Hospital Universitari Son Espases, Palma de Mallorca, Spain); Ester Vendrell Torra and Jordi Almirall Pujol (Intensive Care Medicine, Hospital de Mataró, Spain). South Africa—Charles Feldman (Division of Pulmonology, Department of Internal Medicine, Charlotte Maxeke Johannesburg Academic Hospital, Faculty of Health Sciences, University of the Witwatersrand, Johannesburg, South Africa). South Korea—Ho Kee Yum (Inje Univ. Seoul Paik Hospital, South Korea). Togo—Arnauld Attannon Fiogbe (Pulmonology and Infectious Diseases Service/University hospital of Sylvanus Olympio, Lomé, Togo). Tunisia—Ferdaous Yangui (Department of Pneumology, Hospital of Internal Forces Security (I.F.S), Marsa, Tunis, Tunisia). Turkey—Semra Bilaceroglu (Izmir Dr. Suat Seren Training and Research Hospital for Thoracic Medicine and Surgery, Izmir, Turkey); Levent Dalar (Pulmonary Medicine, Istanbul Bilim University, Istanbul, Turkey); Ufuk Yilmaz (Suat Seren Chest Disease and Surgery Training and Research Hospital, İzmir, Turkey). Ukraine—Artemii Bogomolov (Vinnitsa National Pirogov Memorial Medical University, Vinnitsa regional antituberculosis hospital, Vinnitsa, Ukraine). United Arab Emirates—Naheed Elahi (Dubai Hospital, UAE.); UK—Devesh J Dhasmana (Victoria Hospital, Kirkcaldy, NHS Fife, UK); Rhiannon Ions, Julie Skeemer, and Gerrit Woltmann (University Hospitals of Leicester NHS Trust and University of Leicester, Leicester, UK); Carole Hancock (Royal Respiratory Research Team, Royal Liverpool University Hospital, Liverpool, UK); Adam T Hill (Royal Infi rmary and University of Edinburgh, UK); Banu Rudran (The Royal London Hospital, Barts Health Trust, London, UK); Silvia Ruiz- Buitrago and Marion Campbell (Hairmyres Hospital, Eaglesham Road, East Kilbride, UK); Paul Whitaker (Department of Respiratory Medicine, St James’s Hospital, Leeds, UK). USA—Karen S Allen (University of Oklahoma Health Sciences Center, OK, USA); Veronica Brito (Texas A&M Health Science Center, Division of Pulmonary, Critical Care and Sleep Medicine Baylor Scott & White Health, TX, USA); Jessica Dietz (Fargo VA Health Care System, Fargo, ND, USA); Claire E Dysart and Susan M Kellie (Clement J Zablocki VA Medical Center, Milwaukee, WI, USA, Division of Infectious Diseases, University of New Mexico School of Medicine, Raymond G Murphy VA Medical Center, Albuquerque, NM, USA); Ricardo A Franco-Sadud and Garnet Meier (Division of Hospital Medicine, Cook County Hospital, Chicago, MI, USA); Mina Gaga (7th Resp Med Dept and Asthma Center, Athens Chest Hospital, USA); Thomas L Holland and Stephen P Bergin (Department of Medicine, Duke University Medical Center and School of Medicine, Duke Clinical Research Institute, NC, USA); Fayez Kheir (Department Pulmonary Diseases, Critical Care & Environmental Medicine, Tulane University Health Sciences Center, New Orleans, LA, USA); Mark Landmeier (Division of Pulmonary and Critical Care Medicine, Northwestern Memorial Hospital, Chicago, IL, USA); Manuel Lois (John Peter Smith Hospital, Fort Worth, TX, USA); Girish B Nair (Interstitial Lung Disease Program and Pulmonary Rehabilitation, SUNY Stony Brook Winthrop University Hospital, Mineola, NY, USA); Hemali Patel (Department of Medicine, Division of General Internal Medicine, Hospital Medicine Group, University of Colorado, USA); Katherine Reyes (Henry Ford Hospital, Detroit, IL, USA); William Rodriguez-Cintron (Pulmonary/Critical Care Medicine VA Caribbean Healthcare System, USA); Shigeki Saito (Tulane University, New Orleans, USA); Nilam J Soni, Julio Noda, Cecilia I Hinojosa, Stephanie M Levine, Luis F Angel, and Antonio Anzueto (Divisions of Hospital Medicine & Pulmonary/Critical Care Medicine, South Texas Veterans Health Care System, University of Texas Health Science Center San Antonio, San Antonio, TX, USA); K Scott Whitlow, John Hipskind, and Kunal Sukhija (Kaweah Delta Health Care District, Department of Emergency Medicine, Visalia, CA, USA); Richard G. Wunderink and Ray D Shah (Northwestern University Feinberg School of Medicine, Chicago, IL, USA). Zambia—Kondwelani John Mateyo (Department of Internal Medicine, University Teaching Hospital, Lusaka, Zambia).

## References

[1] Gramegna (2018). Atypical pathogens in hospitalized patients with community-acquired pneumonia: a worldwide perspective. BMC Infect Dis.

