# Supplementary material for: Atypical pathogens in hospitalized patients with community-acquired pneumonia: a worldwide perspective
Source: BMC Infect Dis. 2018 Dec 18;18:677. doi: 10.1186/s12879-018-3565-z (PMC6299604; doi:10.1186/s12879-018-3565-z)
Supplement: Supplementary file 1 — Table A: Tables with testing frequencies for specific atypical pathogens across continents (A1: Testing frequencies for C. pneumoniae across continents; A2: Testing frequencies for M. pneumoniae across continents; A3: Testing frequencies for L. pneumophila across continents).- Brief description of the data: a table in three parts reporting data about frequency of testing for different atypical pathogens across different continents. (DOC 50 kb) [file 12879_2018_3565_MOESM1_ESM.doc]

**ADDITIONAL FILES**

**Table A: Tables with testing frequencies for specific atypical pathogens across continents**

**A1: Testing frequencies for *C. pneumoniae*** across continents

| **Continent** | **Tested/Total (%)** | **Rest of the world**  **Tested/Total (%)** | **P** |
| --- | --- | --- | --- |
| Europe | 202/2344 (8.6%) | 26/1358 (1.9%) | <0.0001 |
| South America | 11/218 (5%) | 217/3484 (6.2%) | 0.48 |
| Africa | 6/156 (3.8%) | 222/3546 (6.3%) | 0.22 |
| Oceania | 1/40 (2.5%) | 227/3662 (6.2%) | 0.51 |
| North America | 5/529 (0.9%) | 223/3173 (7%) | <0.0001 |
| Asia | 3/415 (0.7%) | 225/3287 (6.8%) | <0.0001 |

**A2: Testing frequencies for *M. pneumoniae*** across continents

| **Continent** | **Tested/Total (%)** | **Rest of the world**  **Tested/Total (%)** | **P** |
| --- | --- | --- | --- |
| Europe | 209/2344 (8.9%) | 42/1358 (3.1%) | <0.0001 |
| Oceania | 3/40 (7.5%) | 248/3662 (6.8%) | 0.75 |
| South America | 10/218 (4.6%) | 241/3484 (6.9%) | 0.18 |
| Asia | 14/415 (3.4%) | 237/3287 (7.2%) | 0.003 |
| North America | 13/529 (2.5%) | 238/3173 (7.5%) | <0.0001 |
| Africa | 2/156 (1.3%) | 249/3564 (7%) | 0.005 |

**A3: Testing frequencies for *L. pneumophila*** across continents

| **Continent** | **Tested/Total (%)** | **Rest of the world**  **Tested/Total (%)** | **P** |
| --- | --- | --- | --- |
| Europe | 1041/2344 (44.4%) | 145/1358 (10.7%) | <0.0001 |
| North America | 102/529 (19.3%) | 1084/3173 (34.2%) | <0.0001 |
| Asia | 35/415 (8.4%) | 1151/3287 (35.2%) | <0.0001 |
| Oceania | 2/40 (5%) | 1184/3662 (34%) | <0.0001 |
| Africa | 4/156 (2.6%) | 1182/3546 (33%) | <0.0001 |
| South America | 2/218 (0.9%) | 1184/3484 (34%) | <0.0001 |
